# Genome editing–induced t(4;11) chromosomal translocations model B cell precursor acute lymphoblastic leukemias with KMT2A-AFF1 fusion

**DOI:** 10.1172/JCI171030

**Published:** 2024-01-02

**Authors:** Feng Pan, Jolanda Sarno, Johan Jeong, Xin Yang, Astraea Jager, Tanja A. Gruber, Kara L. Davis, Michael L. Cleary

**Affiliations:** 1 Department of Pathology, Stanford University School of Medicine, Stanford, California, USA.; 2 Department of Molecular Medicine, the University of Texas Health Science Center at San Antonio, San Antonio, Texas, USA.; 3 Division of Pediatric Hematology, Oncology, Stem Cell Transplantation and Regenerative Medicine, Stanford University School of Medicine, Stanford, California, USA.; 4 Stanford Center for Cancer Cell Therapy, Stanford Cancer Institute, Stanford University, Stanford, California, USA.

**Keywords:** Hematology, Oncology, Leukemias, Mouse models

## Abstract

A t(4;11) leukemia model established from CRISPR-engineered chromosomal translocations between the KMT2A and AFF1 genes recapitulate proteomic, epigenomic, and transcriptomic features of primary patient leukemias.

**To the Editor:** The (t;411) (q21q23) chromosomal translocation fuses lysine methyltransferase 2A (*KMT2A*) to ALF transcription elongation factor 1 (*AFF1*), the most common KMT2A fusion partner, and is prevalent in B cell precursor acute lymphoblastic leukemia (BCP-ALL) in both adults and children (1). Recently, CRISPR-mediated *KMT2A* rearrangement (KMT2Ar) in human umbilical cord blood (UCB) hematopoietic stem and progenitor cells (HSPCs) was used to model aspects of leukemia biology (2–4). Here, we induced chromosomal translocations between the *KMT2A* and *AFF1* genes in primary human UCB HSPCs to model (t;411) leukemia and performed multiomics analyses on the resultant gene-edited BCP-ALLs in comparison with patient ALLs and normal bone marrow from healthy donors (Figure 1A).

CRISPR/Cas9 ribonucleoprotein complexes targeting intronic break point cluster regions in the *KMT2A* and *AFF1* genes, respectively, were introduced into primary human UCB HSPCs to generate *KMT2A-AFF1* gene fusions that mimic those in human ALLs (Figure 1B and Supplemental Figure 1, A–C; supplemental material available online with this article; https://doi.org/10.1172/JCI171030DS1). At various times of culture, gene-edited cells were transplanted into sublethally irradiated, immunodeficient mice, which succumbed to lethal hematological malignancies with latencies of 6 to 9 months, versus no abnormalities in Cas9 control mice (Figure 1C and Supplemental Figure 1, D–H). Flow cytometry showed leukemia cell phenotypes characteristic of BCP-ALL (CD19^+^CD33^–^) (Supplemental Figure 1F). The observed B lineage skewing occurred despite culture in myeloid-conditioned medium prior to transplantation, suggesting an instructive role of KMT2A-AFF1 and the reciprocal fusion in B lineage commitment in this model. Leukemia lineage and disease features were conserved in secondary transplant recipients but with accelerated onset (Figure 1C and Supplemental Figure 1I). Contrary to the oligoclonal composition of in vitro–cultured cells, monoclonal *KMT2A-AFF1* break point sequences observed in primary BCP-ALLs indicated that gene-edited cells underwent selection for clonal leukemias in vivo (Supplemental Figure 1J).

To investigate the ontology of the gene-edited (t;411) BCP-ALL cells, we performed single-cell mass cytometry and applied a B cell developmental classifier, as previously developed (5). This analysis revealed that *KMT2A-AFF1* gene-edited cells were arrested in an early stage of B cell development, specifically at the pre-pro-B cell stage, when compared with engrafted Cas9 control cells (Supplemental Figure 1K). Cells harboring *KMT2A-AFF1* translocation were classified in a less differentiated B cell stage compared with primary samples with other prognostic translocations, corroborating prior findings (Figure 1D) (5). When we compared classified leukemia cell subsets with their normal B cell counterparts, high-dimensional phenotypes showed that gene-edited (t;411) BCP-ALL shared t-distributed stochastic neighbor embedding (t-SNE) space with corresponding patient BCP-ALL and PDX samples (Figure 1E) but not human leukemia cell lines (Supplemental Figure 2A), suggesting that the *KMT2A-AFF1* gene-edited cells overall recapitulate the same phenotype and intracellular state of primary BCP-ALL cells harboring (t;411) translocation. Despite high expression of common BCP-ALL–associated proteins, such as CD19, CD34, and CD38, expression of others, including CD133, PAX5, and BCL2, was more heterogeneous (Supplemental Figure 2, B–D), indicating the phenotypic heterogeneity of (t;411) BCP-ALL. Consistent with previous studies (6, 7), *KMT2A-AFF1* gene-edited cells were greatly restricted by the immature negative CD10 expression (Supplemental Figure 2C).

Correlation analysis of genome-wide chromatin accessibility and RNA-Seq indicated distinct clustering of samples (Figure 1, F and G). Gene-edited (t;411) cells, (t;911) ALL cells (4), and patient KMT2A-AFF1 BCP-ALL grouped together more than other leukemia subsets (gene-edited KMT2A-MLLT3 AML, MPAL, and KMT2A-AFF1 human cell lines), consistent with their phenotypic similarities. Notably, epigenomic and transcriptomic profiles of human cell lines MV4;11, SEM, and RS4;11 differed from those of the gene-edited leukemias and primary patient samples (Supplemental Figure 3, A–C), again suggesting that human KMT2A-AFF1 cell lines are poor surrogates for primary leukemia biology. The association between KMT2A-AFF1 BCP-ALL chromatin accessibility landscapes and gene expression programs was then assessed, which identified 17,756 accessible regions corresponding to 2,343 genes. These genes included established KMT2Ar and lineage-specific signatures, such as *MEIS1*, *CDKN2A*, and *BCL11A*. The group was enriched for specific gene ontology terms, including B cell receptor complex, Bcl-2 family protein complex, and acute lymphoblastic leukemia (Supplemental Figure 3D).

In summary, CRISPR editing in human HSPCs generates de novo (t;411) leukemia that captures the phenotypic, transcriptional, and chromatin accessibility signatures of human KMT2A-AFF1 BCP-ALL, highlighting the advantages of gene-edited cells for modeling human disease. KMT2A-AFF1 drives distinct lymphoid gene expression programs, leading to a developmental block at the earliest stages of B cell development. Although this model may not fully recapitulate leukemias that develop in patients, particularly under immune surveillance, it can improve understanding of the pathogenesis of KMT2A-AFF1 BCP-ALL and facilitate the development of novel therapeutic and diagnostic approaches.

## Supplementary Material

Supplemental data

Supporting data values

## Figures and Tables

**Figure 1:**
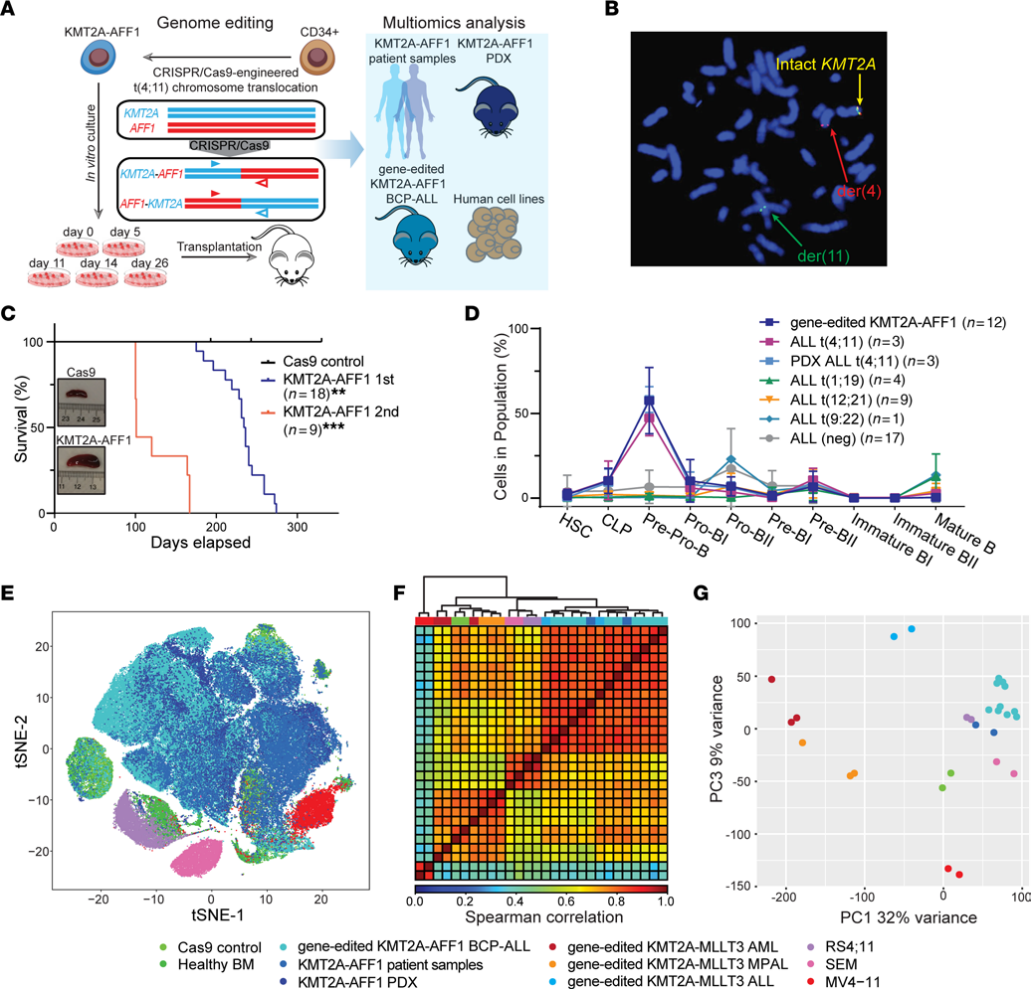
CRISPR-engineered KMT2A-AFF1 BCP-ALLs recapitulate proteomic, epigenomic, and transcriptomic features of primary patient leukemias. (**A**) Schematic of the phenotypic, genetic, and epigenetic characterization of KMT2A-AFF1 cells generated through CRISPR/Cas9-mediated gene editing. PDX, patient-derived xenograft. (**B**) FISH analysis of cells (day 26 in vitro culture) for KMT2A translocation using *KMT2A* break apart probes. (**C**) Survival curves for xeno-transplanted mice showing mean latencies for development of primary (*n* = 18) and secondary (*n* = 9) leukemias, respectively. *P* value was generated using Mantel-Cox log-rank test. **P < 0.01; ***P < 0.001. (**D**) Percentage of cells from gene-edited KMT2A-AFF1 BCP-ALL and diagnostic KMT2A-AFF1 BCP-ALL patient bone marrow classified into developmental populations. HSC, hematopoietic stem cell; CLP, common lymphoid progenitor. (**E**) t-SNE projections of normal controls, gene-edited BCP-ALL, patient samples, and PDX and human cell lines, where each cell is represented by a dot within color-coded clusters. (**F**) Unsupervised hierarchical clustering of Spearman’s correlations from ATAC-Seq data of gene-edited KMT2A-AFF1 BCP-ALLs, gene-edited KMT2A-MLLT3 leukemias (acute myeloid leukemia [AML], ALL, mixed-phenotype acute leukemia [MPAL]), KMT2A-AFF1 patient samples, and KMT2A-AFF1 human cell lines. (**G**) Principal component analysis of RNA-Seq data from gene-edited BCP-ALLs, gene-edited leukemias (AML, ALL, MPAL), patient samples, and human cell lines.

## Version 1 11/02/2023

In-Press Preview

## Version 2 01/02/2024

Electronic publication
