# Supplementary material for: Genome editing–induced t(4;11) chromosomal translocations model B cell precursor acute lymphoblastic leukemias with KMT2A-AFF1 fusion
Source: J Clin Invest. 2024 Jan 2;134(1):e171030. doi: 10.1172/JCI171030 (PMC10760968; doi:10.1172/JCI171030)
Supplement: Supplemental data [file jci-134-171030-s083.pdf]

Supplemental Data

Genome editing-induced t(4;11) chromosomal translocations model B cell precursor acute lymphoblastic leukemias with KMT2A-AFF1 fusion

Feng Pan[1,2], Jolanda Sarno[3,4], Johan Jeong[1], Xin Yang[1], Astraea Jager[3,4], Tanja A. Gruber[3], Kara L. Davis[3,4], and Michael L. Cleary[1]

[1]Department of Pathology, Stanford University, Stanford, CA
[2]Department of Molecular Medicine, the University of Texas Health Science Center at San Antonio, San Antonio, TX
[3]Department of Pediatrics, Hematology, Oncology, Stem Cell Transplant and Regenerative Medicine, Stanford University School of Medicine, Stanford, CA
[4]Stanford Center for Cancer Cell Therapy, Stanford Cancer Institute, Stanford University, Stanford, CA

Corresponding Author:
Michael L. Cleary, MD
Lokey Stem Cell Research Building, Room G2034
Stanford, CA 94305
Phone: 650-723-5471
Email: mcleary@stanford.edu

Contents:
Supplemental Figures 1-3
Methods
References

Figure S1

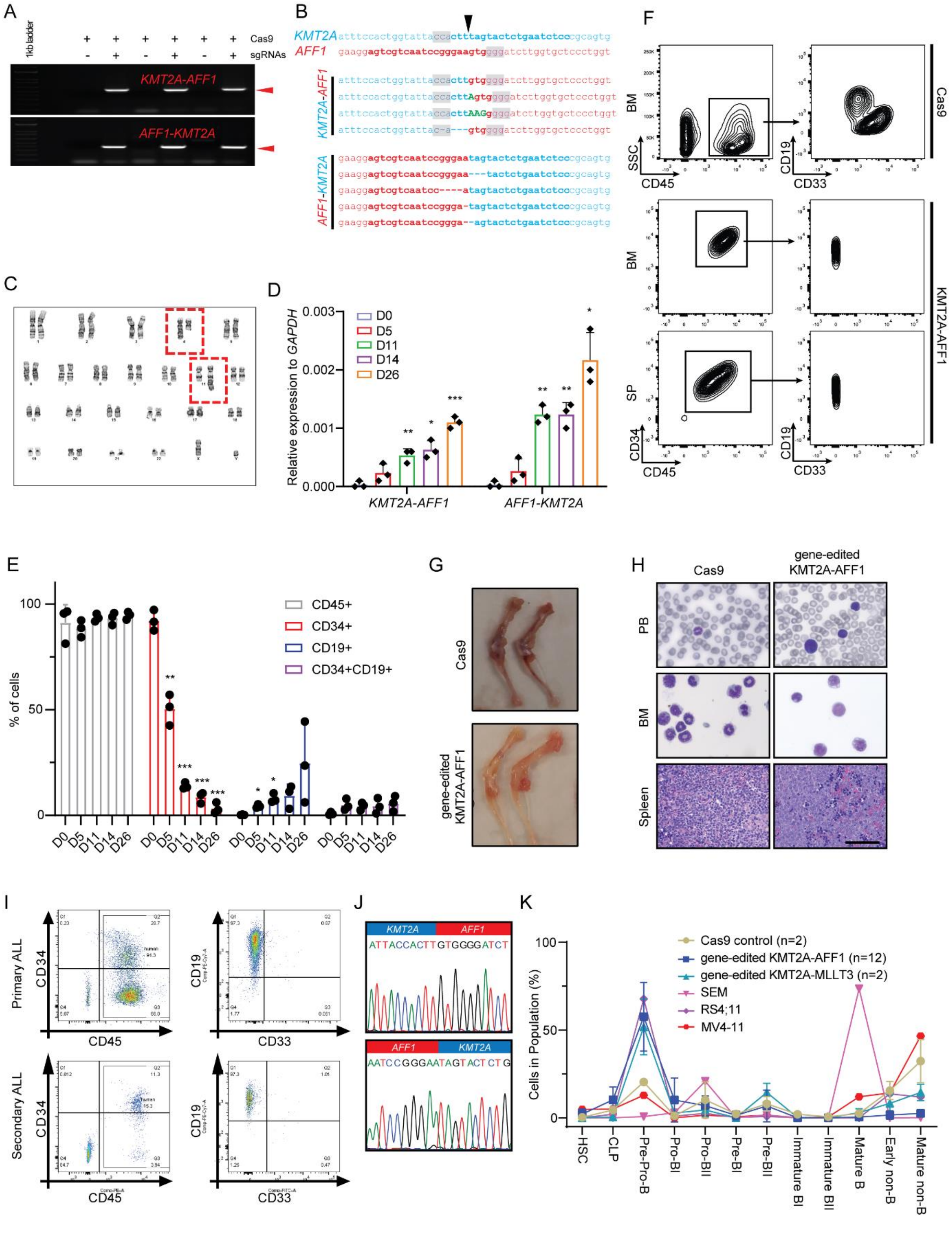
A
1kb ladder
Cas9
sgRNAs
KMT2A-AFF1
AFF1-KMT2A
B
KMT2A
AFF1
KMT2A-AFF1
AFF1-KMT2A
C
D
Relative expression to GAPDH
D0
D5
D11
D14
D26
KMT2A-AFF1
AFF1-KMT2A
F
BM
SP
SSC
CD45
CD19
CD33
CD34
Cas9
KMT2A-AFF1
E
% of cells
CD45+
CD34+
CD19+
CD34+CD19+
G
Cas9
gene-edited KMT2A-AFF1
H
Cas9
gene-edited KMT2A-AFF1
PB
BM
Spleen
I
Primary ALL
Secondary ALL
CD34
CD45
CD19
CD33
J
KMT2A
AFF1
ATTACCACTTGTGGGGATCT
AATCCGGGAATAGTACTCTG
K
Cells in Population (%)
Cas9 control (n=2)
gene-edited KMT2A-AFF1 (n=12)
gene-edited KMT2A-MLLT3 (n=2)
SEM
RS4;11
MV4-11
HSC
CLP
Pre-Pro-B
Pro-BI
Pro-BII
Pre-BI
Pre-BII
Immature BI
Immature BII
Mature B
Early non-B
Mature non-B

Figure S1: High-efficiency induction of t(4;11) chromosomal translocations.

(A) Expression of *KMT2A-AFF1* and reciprocal *AFF1-KMT2A* transcripts (red arrowheads) were detected by RT-PCR on cDNA from 3 independent KMT2Ar cell cultures.

(B) Genomic DNA sequences of *KMT2A-AFF1* and *AFF1-KMT2A* junctions in gene-edited cells. sgRNA and PAM sequences are denoted by bold type and gray shading, respectively. ▼, Cas9 cutting position.

(C) Representative karyotype with KMT2A-AFF1 translocation.

(D) RT-qPCR of *KMT2A-AFF1* and *AFF1-KMT2A* transcripts from CRISPR-engineered KMT2A-AFF1 cultures over time. Data are representative of three biological replicates and error bars represent SD of the mean. *$p < 0.05$; **$p < 0.01$; ***$p < 0.001$. unpaired Student's t test.

(E) Quantification of human cell immunophenotypes in CRISPR-engineered KMT2A-AFF1cultures over time. Data are representative of three biological replicates and error bars represent SD of the mean. *$p < 0.05$; **$p < 0.01$; ***$p < 0.001$. unpaired Student's t test.

(F) Flow cytometry analysis of tissues from representative leukemic mice for myeloid (CD33) and lymphoid (CD19) lineage markers.

(G) Representative images of the leg bones from Cas9 control and KMT2A-AFF1 mice.

(H) Leukemic involvement of peripheral blood (PB), bone marrow (BM), and spleen from Cas9 control and KMT2A-AFF1 primary recipient mice. Scale bar = 2.5µm.

(I) Flow cytometry analysis of a KMT2Ar primary ALL bone marrow cells and their respective secondary transplants.

(J) Sanger sequencing tracks showing *KMT2A-AFF1* and *AFF1-KMT2A* genomic DNA breakpoints in blasts isolated from the bone marrow of KMT2A-AFF1 mice.

(K) Percentage of cells from Cas9 control (n = 2), gene-edited KMT2A-AFF1 BCP-ALL (n = 12), and human cell lines classified into each developmental population.

Figure S2

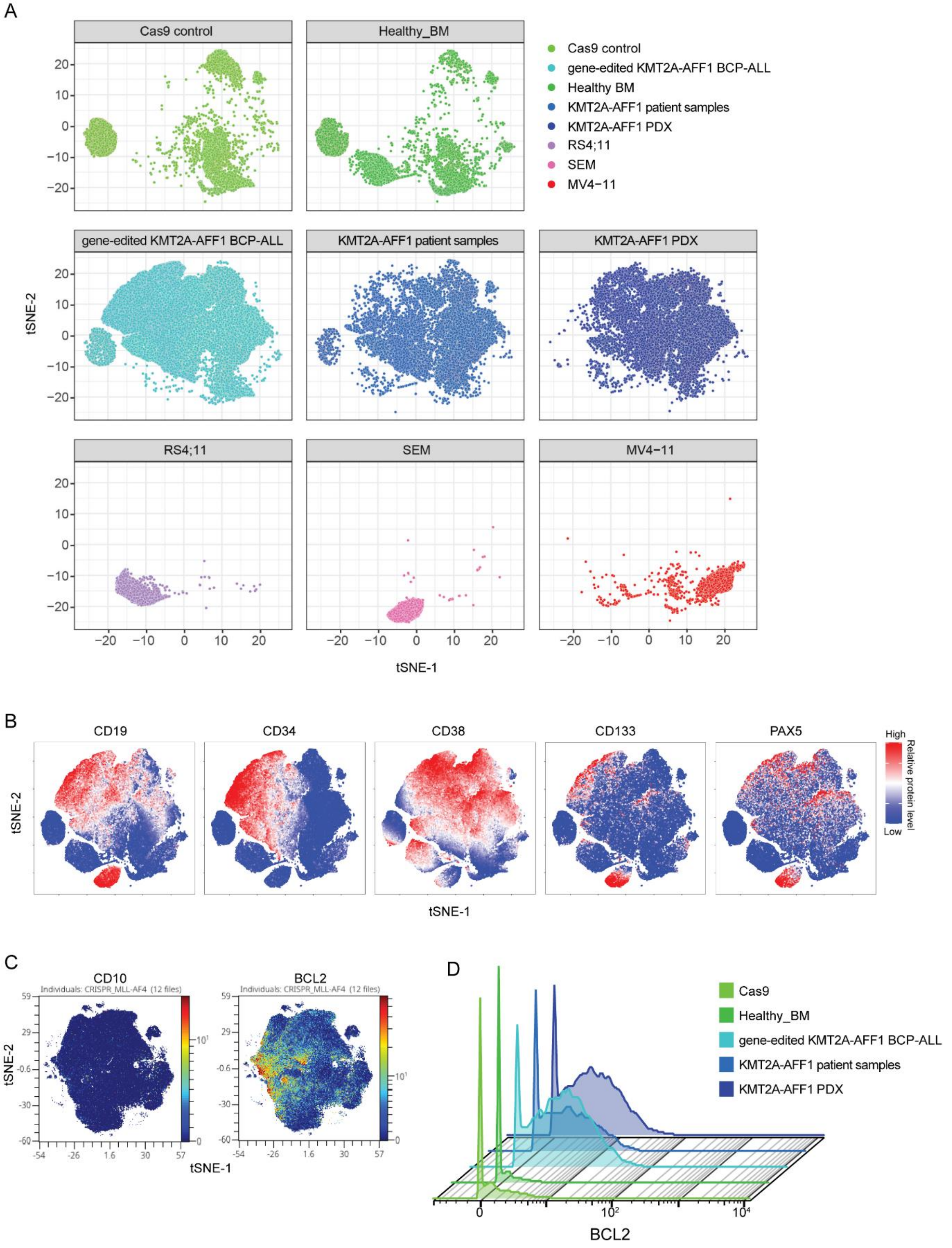
A
Cas9 control
Healthy_BM
Cas9 control
gene-edited KMT2A-AFF1 BCP-ALL
Healthy BM
KMT2A-AFF1 patient samples
KMT2A-AFF1 PDX
RS4;11
SEM
MV4−11
gene-edited KMT2A-AFF1 BCP-ALL
KMT2A-AFF1 patient samples
KMT2A-AFF1 PDX
RS4;11
SEM
MV4−11
tSNE-2
tSNE-1
B
CD19
CD34
CD38
CD133
PAX5
High
Low
Relative protein level
tSNE-2
tSNE-1
C
CD10
BCL2
Individuals: CRISPR_MLL-AF4 (12 files)
tSNE-2
tSNE-1
D
Cas9
Healthy_BM
gene-edited KMT2A-AFF1 BCP-ALL
KMT2A-AFF1 patient samples
KMT2A-AFF1 PDX
BCL2

Figure S2: CyTOF analysis of leukemias induced by gene-edited HSPCs.

(A) Example of tSNE analysis from the leukemia compartment as identified in (Fig. 1E).

(B) Expression of CD19, CD34, CD38, CD133, and PAX5 from normal and tumor tissues.

(C) Expression of CD10 and BCL2 from normal and tumor tissues.

(D) Histogram of BCL2 expression analyzed in normal and tumor tissues.

Figure S3

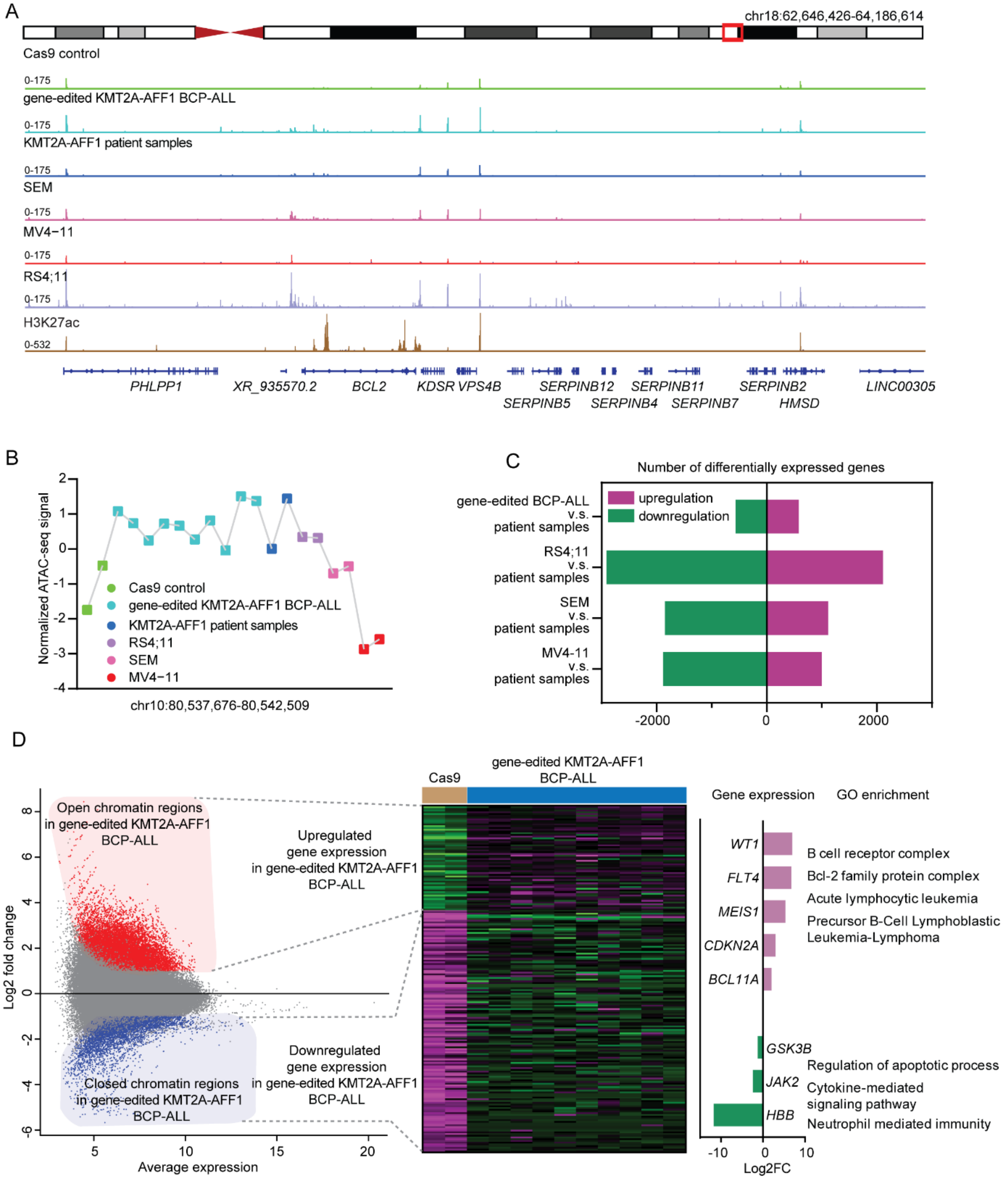
A
chr18:62,646,426-64,186,614
Cas9 control
0-175
gene-edited KMT2A-AFF1 BCP-ALL
0-175
KMT2A-AFF1 patient samples
0-175
SEM
0-175
MV4−11
0-175
RS4;11
0-175
H3K27ac
0-532
PHLPP1
XR_935570.2
BCL2
KDSR VPS4B
SERPINB12
SERPINB11
SERPINB2
LINC00305
SERPINB5
SERPINB4
SERPINB7
HMSD
B
Normalized ATAC-seq signal
Cas9 control
gene-edited KMT2A-AFF1 BCP-ALL
KMT2A-AFF1 patient samples
RS4;11
SEM
MV4−11
chr10:80,537,676-80,542,509
C
Number of differentially expressed genes
gene-edited BCP-ALL v.s. patient samples
RS4;11 v.s. patient samples
SEM v.s. patient samples
MV4-11 v.s. patient samples
upregulation
downregulation
-2000
0
2000
D
Open chromatin regions in gene-edited KMT2A-AFF1 BCP-ALL
Closed chromatin regions in gene-edited KMT2A-AFF1 BCP-ALL
Log2 fold change
Average expression
Upregulated gene expression in gene-edited KMT2A-AFF1 BCP-ALL
Downregulated gene expression in gene-edited KMT2A-AFF1 BCP-ALL
Cas9
gene-edited KMT2A-AFF1 BCP-ALL
Gene expression
GO enrichment
WT1
FLT4
MEIS1
CDKN2A
BCL11A
GSK3B
JAK2
HBB
B cell receptor complex
Bcl-2 family protein complex
Acute lymphocytic leukemia
Precursor B-Cell Lymphoblastic Leukemia-Lymphoma
Regulation of apoptotic process
Cytokine-mediated signaling pathway
Neutrophil mediated immunity
Log2FC

Figure S3: Chromatin accessibility and transcriptional landscapes of gene-edited HSPCs display leukemic features.

(A) Normalized ATAC-seq signal profiles at a locus in Cas9 control, gene-edited KMT2A-AFF1 BCP-ALLs, KMT2A-AFF1 patient samples, and KMT2A-AFF1 human cell lines, shown together with a normalized B cell H2K27ac chromatin immunoprecipitation sequencing profile from ENCODE.

(B) Normalized ATAC-seq signal at representative region.

(C) Bar plot showing the number of differentially expressed genes (DEGs; FDR < 0.05) in the indicated comparisons.

(D) Integration of ATAC-seq and RNA-seq links BCP-ALL associated epigenetic and gene expression changes (foldchange >2, FDR<0.05).

Materials and methods

Cell culture

Human leukemia cell lines were obtained from ATCC and maintained in RPMI 1640 with 10% FBS and 1% penicillin-streptomycin. Human CD34$^+$ HSPCs and CRISPR-Cas9 engineered KMT2A-AFF1 leukemia cells were cultured in StemSpan SFEM II medium with SCF (50 ng/mL), thrombopoietin (100 ng/mL), Flt3 ligand (100 ng/mL), IL-6 (100 ng/mL), IL-3 (50 ng/mL), G-CSF (50 ng/mL), UM729 (0.75 µM), StemRegenin 1 (0.75 µM), and 20% FBS at 37°C, 5% CO_2 as previously described (1).

Animals

Gene-edited cells were cultured *in vitro* (day 0 to day 26) and transplanted (1×10^6) by IV injection into sub-lethally irradiated (250 cGy) immune-compromised NOD.Cg PrkdcscidIL2rgtm1Wjl/SzJ (NSG) mice as previously described (1). Four individual primary gene-edited BCP-ALL cells were injected into nine NSG mice to generate the secondary leukemia survival curve.

Human subjects

Human ALL samples were obtained from patients at the Stanford Medical Center with informed consent and institutional review board approval. Human CD34$^+$ HSPCs (three individuals, both male and female) were obtained from Stanford Hospital via the Binns Program for Cord Blood Research under informed consent. Mononuclear cells were purified by Ficoll-density gradient centrifugation enrichment.

CRISPR/Cas9 genome editing

Chemically modified single guide RNAs (sgRNAs) were designed and synthesized by Synthego (Redwood City, CA). The sgRNAs (120 pmol) were incubated with Alt-R® S.p. Cas9 Nuclease V3 (40 pmol, Integrated DNA Technologies, Coralville, IA) for 20 minutes at room temperature prior to electroporation. CD34$^+$ HSPCs (200,000 cells) and the ribonucleoprotein (RNP) complex were resuspended in 20 µL of nucleofection solution and electroporated using a 4D Nucleofector with program DZ-100 (Lonza, Basel, Switzerland).

Chromosomal translocations and fusion gene products detection

Total RNA was extracted as described in RNA-seq. cDNA was synthesized using QuantiTect Reverse Transcription Kit (Qiagen, Hilden, Germany). qRT-PCR was performed using PowerUp SYBR Green Master Mix on CFX96 and quantified using the ddCT.

Fluorescence in situ hybridization (FISH) and karyotyping were performed by the Cytogenetics Laboratory of Stanford Hospital, as previously described (2).

Mass cytometry (CyTOF)

Samples for CyTOF were prepared as described (3) using a 40-parameters antibody panel built in-house. Viably frozen cells were thawed, resuspended at 1 to 2 million cells per milliliter, and fixed in 1.6% paraformaldehyde for 10 minutes at room temperature. Cell Staining Medium (CSM, phosphate-buffered saline [PBS] + 0.5% bovine serum albumin + 0.02% sodium azide), was used to wash cells. Cells were barcoded using 20-plex barcoding plates prepared in-house as previously described (4). To control for staining and batch effect a healthy BM was added to each barcoded plate. Following barcoding, cells were washed with CSM and combined in one single tube. Human TruStain FcX blocking solution was added to the cells at 50 µL/100 µL staining volume. Metal-conjugated antibodies for surface antigens were added to the cells at titrated concentrations and incubated at room temperature on a shaker for 30 minutes. Cells were washed once with CSM before being permeabilized with 100% methanol for 10 minutes at 4°C. Following permeabilization, cells were washed twice and then stained with metal-conjugated antibodies against intracellular antigens for 30 minutes at room temperature on a shaker. After one CSM was, cells were stained overnight with 1:5,000 191Ir/193Ir DNA intercalator (Fluidigm) in PBS with 1.6% PFA at 4°C. Prior to acquisition on a Helios mass cytometer, cells were washed once in CSM, followed by two washes in ultrapure double-distilled H_2O. Cells were resuspended in ddH_2O with normalization beads (139La/142Pr/159Tb/169Tm/175Lu), maintained at 4°C and acquired on a Helios mass cytometer at a rate of ~200 cells per second.

Processing and analysis of CyTOF data

CyTOF raw data were normalized using bead normalization (5) and files were debarcorded as described (4). Cytobank software was used to gate Lin-/B-enriched cells excluding dead cells

(cPARP+), murine cells (mCD45.1+), myeloid (CD33+) and T-cells (CD3+). We then classified each sample using the B-cell developmental classifier previously published (6). The Lin-/B-enriched cells obtained from the healthy BM control were gated in 12 populations as previously described except for the progenitor's populations combined in one single population named CLP (common lymphoid progenitor). Each cell was then classified to the closest (Mahalanobis distance) healthy gated population based on the expression of 10 proteins (CD34, CD38, CD24, TdT, CD179b, CD19, CD20, IgMi, IgMs) used for the manual gating of the healthy BM. Frequency of each classified sample was then plotted using GraphPad Prism software (v 9.3.1). tSNE dimension reduction analysis was performed by Cytobank and cytofkit2 (https://github.com/JinmiaoChenLab/cytofkit2) using all 40 markers measured at single cell level by mass cytometry.

RNA-seq analysis

RNA was extracted from control and leukemia cells with RNeasy Plus Mini Kit (Qiagen, Hilden, Germany) according to the manufacturer's instructions. RNA-seq libraries were generated and sequenced by Novogene (Sacramento, CA). Bulk RS4;11 RNA-seq datasets are available from GEO under accession number GSE76931. RNA-seq reads were mapped with STAR against the human genome (hg19). Differential expression analysis and Visualization were carried out with raw counts using DESeq2 (7) and iDEP (8).

ATAC-seq analysis

ATAC-seq was performed as described (9). Briefly, control and tumor cells were lysed in cold lysis buffer (10 mM Tris-HCl, pH 7.4, 10 mM NaCl, 3 mM $MgCl_2$, and 0.1% IGEPAL CA-630). Nuclei were centrifuged at 500 × *g* for 10 min, 4°C. Nuclei extract were then incubated with Nextera Tn5 Transposase, 2× TD buffer, and nuclease free water at 37°C for 30 min with gentle mixing. After DNA purification with the MinElute PCR Purification Kit (Qiagen), PCR was performed to amplify the library for additional cycles distinctively according to a quantitative PCR reaction for optimum cycles. The final libraries were sequenced by Novogene. Bulk RS4;11 ATAC-seq datasets are available through EMBL-EBI ArrayExpress under accession number E-MTAB-8676. Adapter sequences were trimmed and reads were mapped to Hg19 using Bowtie2

(10). ATAC-seq peak calling was performed with Genrich (https://github.com/jsh58/Genrich) with default setting.

Statistics

Data are presented as mean ± standard deviation, unless otherwise indicated. Student *t* test (unpaired, 2-tailed) was used to assess significance between 2 groups. Survival curves were analyzed by log-rank (Mantel-Cox) test. $P < .05$ were considered significant. Generation of plots and statistical analyses were performed using Prism GraphPad version 8.

Study Approval

Human samples. Fresh human umbilical cord blood (hUCB) was obtained from Stanford Hospital via the Binns Program for Cord Blood Research under informed consent. ALL patient samples were obtained from the Stanford Medical Center and Lucile Packard Children's Hospital with informed consent and institutional review board approval.

Animal studies. All experiments using mice were performed with the approval of, and in accordance with, the Stanford University Administrative Panel on Laboratory Animal Care.

Data availability

The accession number for the RNA-seq and ATAC-seq data reported in this paper is NCBI GEO: GSE228298. All other data generated or analyzed during this study are included in this published article and values for all data points in graphs are reported in the Supporting Data Values file.

Authorship contributions

FP and MLC conceived the project and wrote the manuscript. FP designed and performed experiments, and analyzed data; JS, JJ, XY, and AJ performed experiments; TAG provided key reagents; JS and KLD interpreted data; MLC supervised the entire project. All authors read and approved the final manuscript.

Acknowledgements

We thank members of the Cleary lab for helpful discussions. This work was supported by fundings from the National Institutes of Health (CA214888) to M.L.C., Alex's Lemonade Stand Foundation to F.P., and Stanford Maternal and Child Health Research Institute (MCHRI) to J.S. and K.L.D.

Conflict of Interest Disclosure

The authors declare no competing financial interests.

Correspondence

Michael L. Cleary, Department of Pathology and Pediatrics, Lokey Stem Cell Research Building, Room G2034, 1291 Welch Rd., Stanford, CA 94305; email, mcleary@stanford.edu

References

1. Jeong J, Jager A, Domizi P, Pavel-Dinu M, Gojenola L, Iwasaki M, et al. High-efficiency CRISPR induction of t(9;11) chromosomal translocations and acute leukemias in human blood stem cells. *Blood Adv.* 2019;3(19):2825-35.
2. Schneidawind C, Jeong J, Schneidawind D, Kim IS, Duque-Afonso J, Wong SHK, et al. MLL leukemia induction by t(9;11) chromosomal translocation in human hematopoietic stem cells using genome editing. *Blood Adv.* 2018;2(8):832-45.
3. Jager A, Sarno J, and Davis KL. Mass Cytometry of Hematopoietic Cells. *Methods Mol Biol.* 2021;2185:65-76.
4. Zunder ER, Finck R, Behbehani GK, Amir el AD, Krishnaswamy S, Gonzalez VD, et al. Palladium-based mass tag cell barcoding with a doublet-filtering scheme and single-cell deconvolution algorithm. *Nat Protoc.* 2015;10(2):316-33.
5. Finck R, Simonds EF, Jager A, Krishnaswamy S, Sachs K, Fantl W, et al. Normalization of mass cytometry data with bead standards. *Cytometry A.* 2013;83(5):483-94.
6. Good Z, Sarno J, Jager A, Samusik N, Aghaeepour N, Simonds EF, et al. Single-cell developmental classification of B cell precursor acute lymphoblastic leukemia at diagnosis reveals predictors of relapse. *Nat Med.* 2018;24(4):474-83.
7. Love MI, Huber W, and Anders S. Moderated estimation of fold change and dispersion for RNA-seq data with DESeq2. *Genome Biol.* 2014;15(12):550.
8. Ge SX, Son EW, and Yao R. iDEP: an integrated web application for differential expression and pathway analysis of RNA-Seq data. *BMC Bioinformatics.* 2018;19(1):534.
9. Buenrostro JD, Wu B, Chang HY, and Greenleaf WJ. ATAC-seq: A Method for Assaying Chromatin Accessibility Genome-Wide. *Curr Protoc Mol Biol.* 2015;109:21 9 1- 9 9.
10. Langmead B, and Salzberg SL. Fast gapped-read alignment with Bowtie 2. *Nat Methods.* 2012;9(4):357-9.
